# Supplementary figures and images for: Genome-wide identification of long non-coding (lncRNA) in Nilaparvata lugens’s adaptability to resistant rice
Source: PeerJ. 2022 Jul 25;10:e13587. doi: 10.7717/peerj.13587 (PMC9332332; doi:10.7717/peerj.13587)

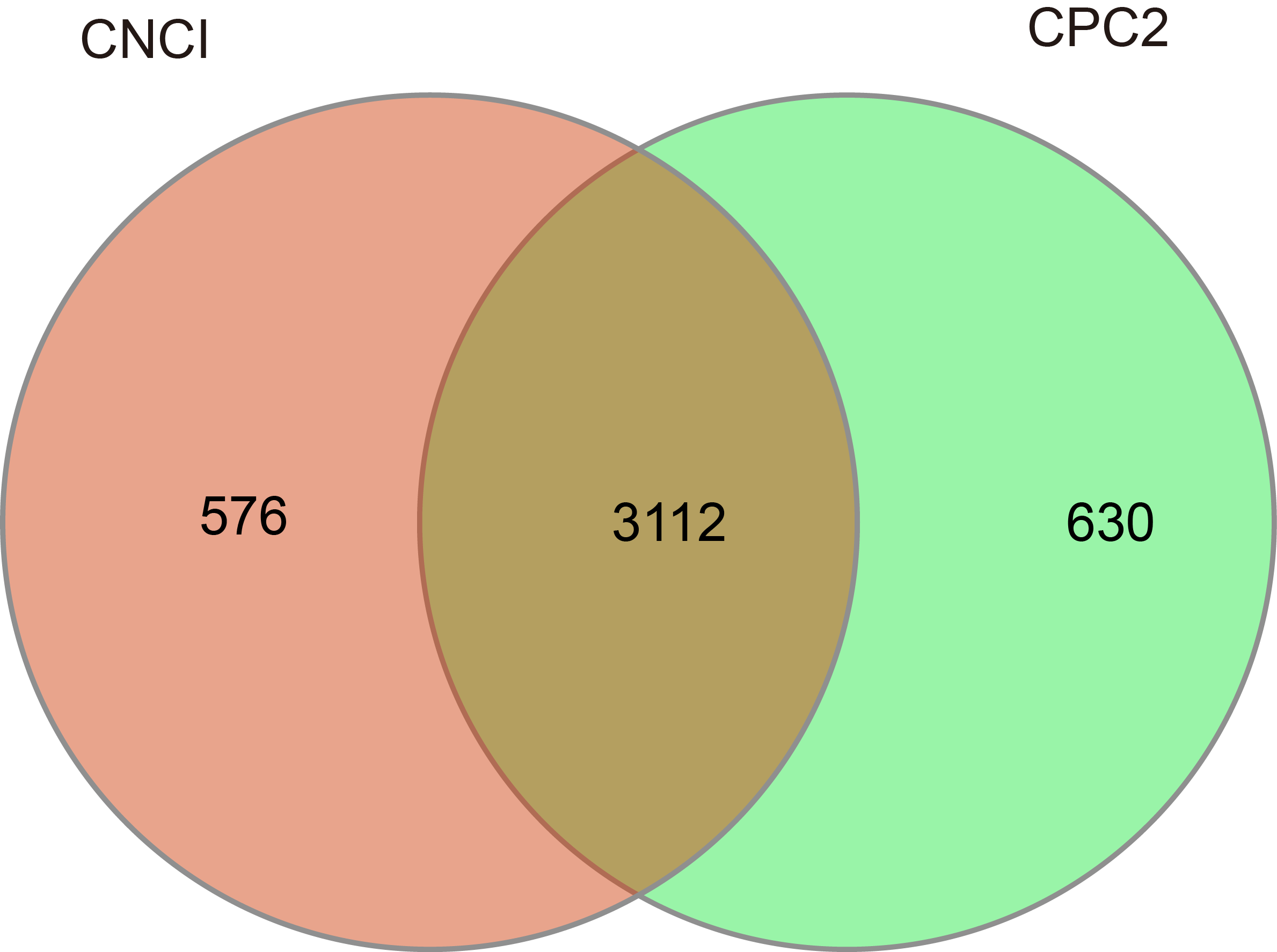

Supplement: Supplemental Information 1 [file peerj-10-13587-s001.png]

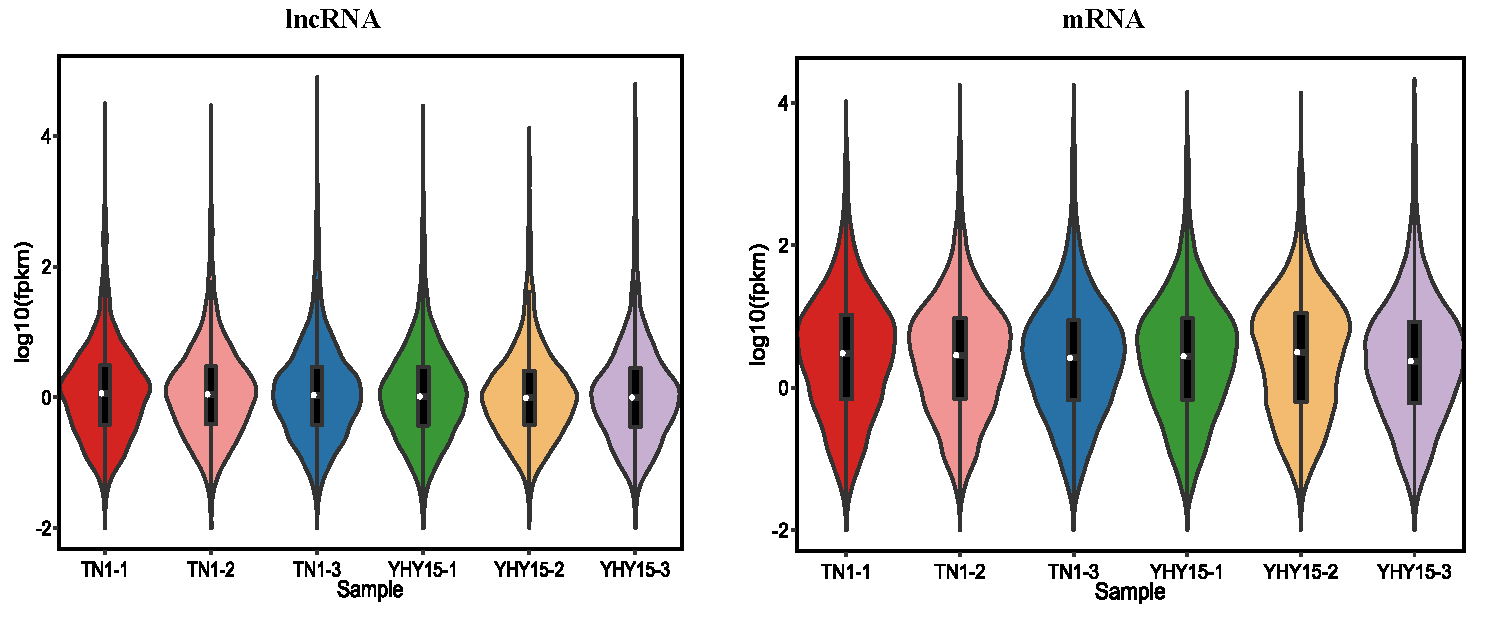

Supplement: Supplemental Information 2 — The ordinate shows the logarithm of the sample expression amount FPKM (fragments per kilobase of transcript per million fragments mapped). [file peerj-10-13587-s002.png]
